# Supplementary material for: Anti-Inflammatory and Tau Phosphorylation–Inhibitory Effects of Eupatin
Source: Molecules. 2020 Nov 30;25(23):5652. doi: 10.3390/molecules25235652 (PMC7731404; doi:10.3390/molecules25235652)

Supplementary figure 1

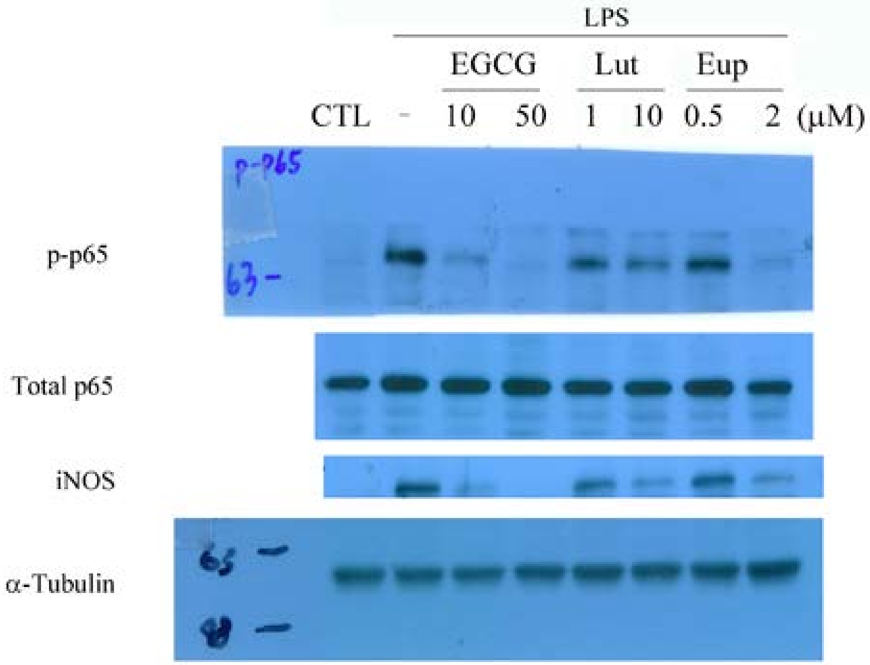

Supplementary figure 2

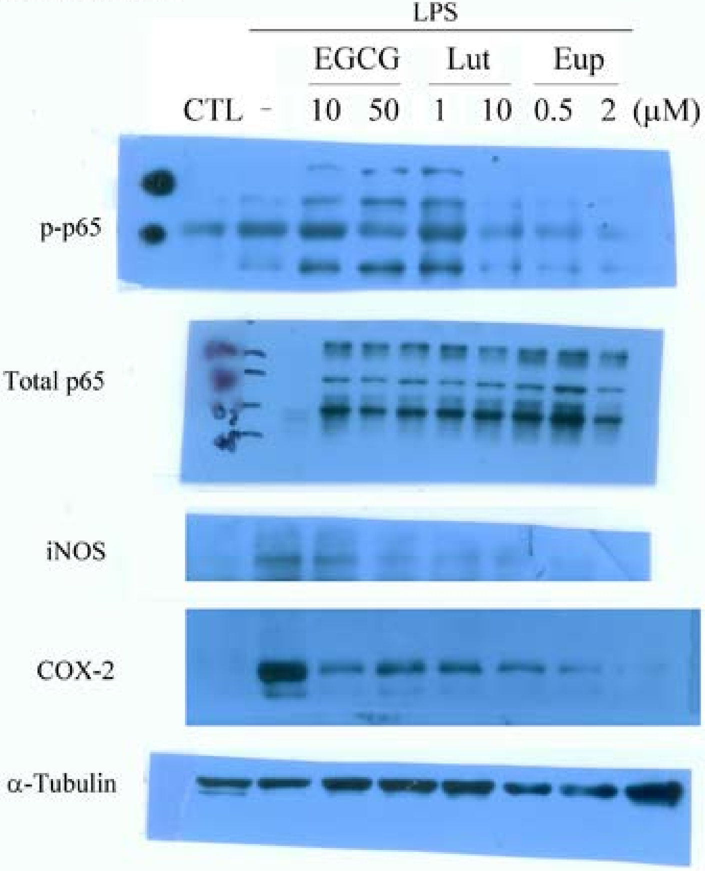

Supplementary figure 3

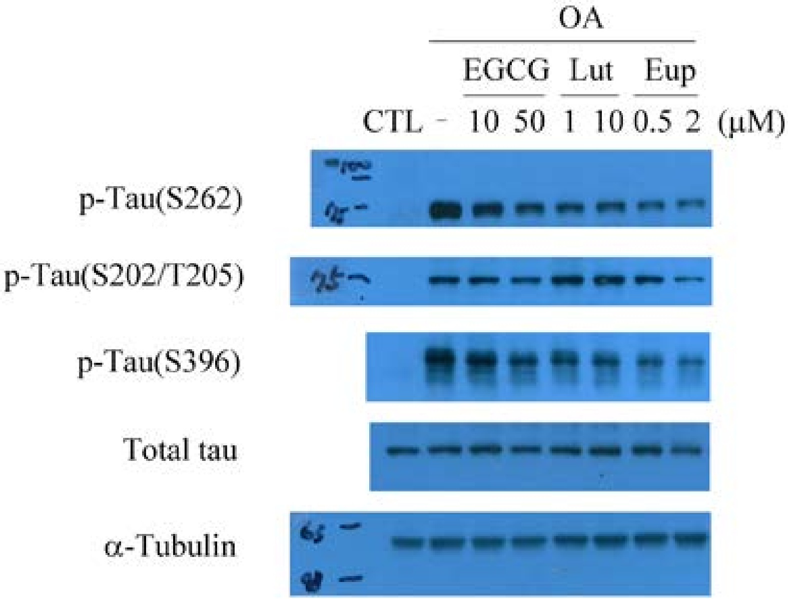

Supplementary figure 4

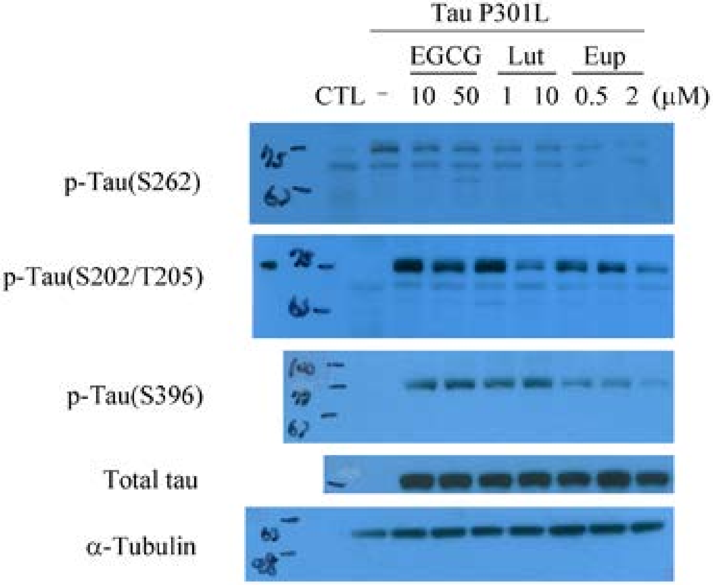

Supplementary figure 5

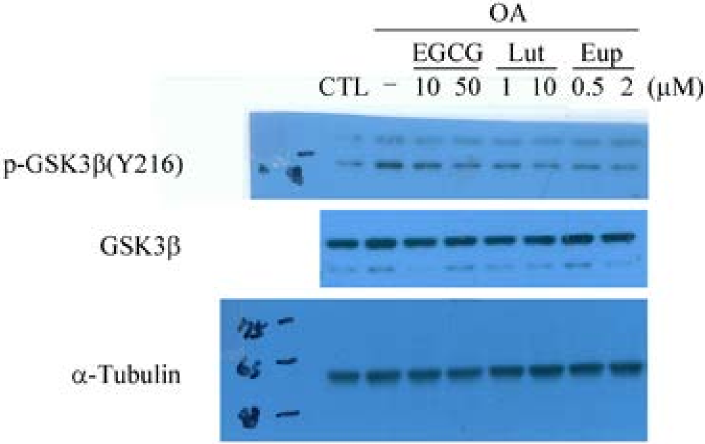

Supplement: Supplementary file 1 [file molecules-25-05652-s001.pdf]
